# The bidirectional relationship between Alzheimer's disease (AD) and epilepsy: A Mendelian randomization study

**DOI:** 10.1002/brb3.3221

**Published:** 2023-09-04

**Authors:** Lianping Xu, Qun Wang

**Affiliations:** ^1^ Department of Neurology, Beijing Tiantan Hospital Capital Medical University Beijing China; ^2^ China National Clinical Research Center for Neurological Diseases Beijing China; ^3^ Beijing Institute of Brain Disorders Collaborative Innovation Center for Brain Disorders Capital Medical University Beijing China

**Keywords:** Alzheimer's disease, Alzheimer's disease (late onset) (load), epilepsy, focal epilepsy, Mendelian randomization

## Abstract

**Background**: There is a complex, bidirectional relationship between Alzheimer's disease (AD) and epilepsy. However, the causality of this association is unclear, as confounders play a role in this association.

**Methods**: We conducted a Mendelian randomization (MR) study to clarify the causal relationship and direction of epilepsy on AD risk. We used publicly available summary statistics to obtain all genetic datasets for the MR analyses. AD and AD‐by‐proxy and late‐onset AD (LOAD) cohorts were included in our study. The epilepsy cohort comprised all epilepsy, generalized epilepsy, focal epilepsy, and its subtypes, as well as some epilepsy syndromes. Next, we conducted validation using another AD cohort.

**Results**: Two correlations between AD and epilepsy using the inverse variance‐weighted (IVW) method are as follows: LOAD and focal epilepsy (OR_IVW_ = 1.079, *p*_IVW_ = .013), focal epilepsy‐documented hippocampal sclerosis (HS) and AD (OR_IVW_ = 1.152, *p*_IVW_ = .017). The causal relationship between epilepsy‐documented HS and AD has been validated (OR_IVW_ = 3.994, *p*_IVW_ = .027).

**Conclusions**: Our MR study provides evidence for a causal relationship between focal epilepsy‐documented HS and AD.

## Graphical abstract

We conducted a bidirectional Mendelian Randomization (MR) study to elucidate the causal relationship and direction between epilepsy and Alzheimer's disease (AD) risk. Our MR study provides evidence for a causal relationship between focal epilepsy‐documented hippocampal sclerosis and AD.

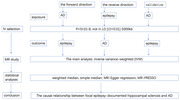

## 1 INTRODUCTION

Epilepsy is a chronic neurological disorder that causes recurrent seizures due to abnormal electrical activity in the brain (Fisher et al., 2017). There are several types of epilepsy, including generalized epilepsy, focal epilepsy, and combined generalized and focal epilepsy. Generalized epilepsy is diagnosed when a patient shows generalized spike‐wave activity on EEG, whereas focal epilepsies involve unifocal or multifocal disorders or seizures involving one hemisphere. Combined generalized and focal epilepsies exist when patients experience “generalized onset” and “focal onset seizures” (Fisher et al., 2014; Scheffer et al., 2017; Thijs et al., 2019). The incidence of epilepsy is highest in infants and the elderly, with several studies showing a peak incidence in the older population, typically rising after the age of 65 (Annegers et al., 1999; Hussain et al., 2006; Sen et al., 2018).

As the population ages, the prevalence of dementia is expected to increase dramatically in the coming decades. In fact, it is projected that by 2050, over 115 million people worldwide will have dementia, with Alzheimer's disease (AD) being the primary cause. AD is a neurodegenerative disorder characterized by the progressive deterioration of cognitive function, including memory, language, and problem‐solving abilities (Alzheimer's Association, 2016). AD can be roughly divided into two subgroups: (1) familial early‐onset cases and (2) late‐onset cases. Late‐onset AD (LOAD) is the most common form of AD and typically occurs in individuals aged 65 and older (Lambert et al., 2013).

The proportion of AD patients with seizures is significantly higher than that of normal elderly people, with a prevalence of about 8% for AD patients with seizures, which increases with the severity of AD (Amatniek et al., 2006). Among individuals with epilepsy, the prevalence of dementia varied between 8.1 and 17.5 per 100 individuals. On the other hand, the pooled prevalence of epilepsy in individuals with dementia was estimated to be 5 per 100 individuals (95% CI 1–9) in population‐based studies and 4 per 100 individuals (95% CI 1–6) in clinic‐based studies. There was limited data available on risk factors and insufficient data to report a pooled overall incidence rate (Subota et al., 2017). A retrospective observational study of 54 patients with amnestic mild cognitive impairment (aMCI)/AD plus epilepsy found that 47% of aMCI/AD‐related seizures were focal with impaired awareness, whereas 55% were nonconvulsive (Vossel et al., 2013).

**FIGURE 1:**
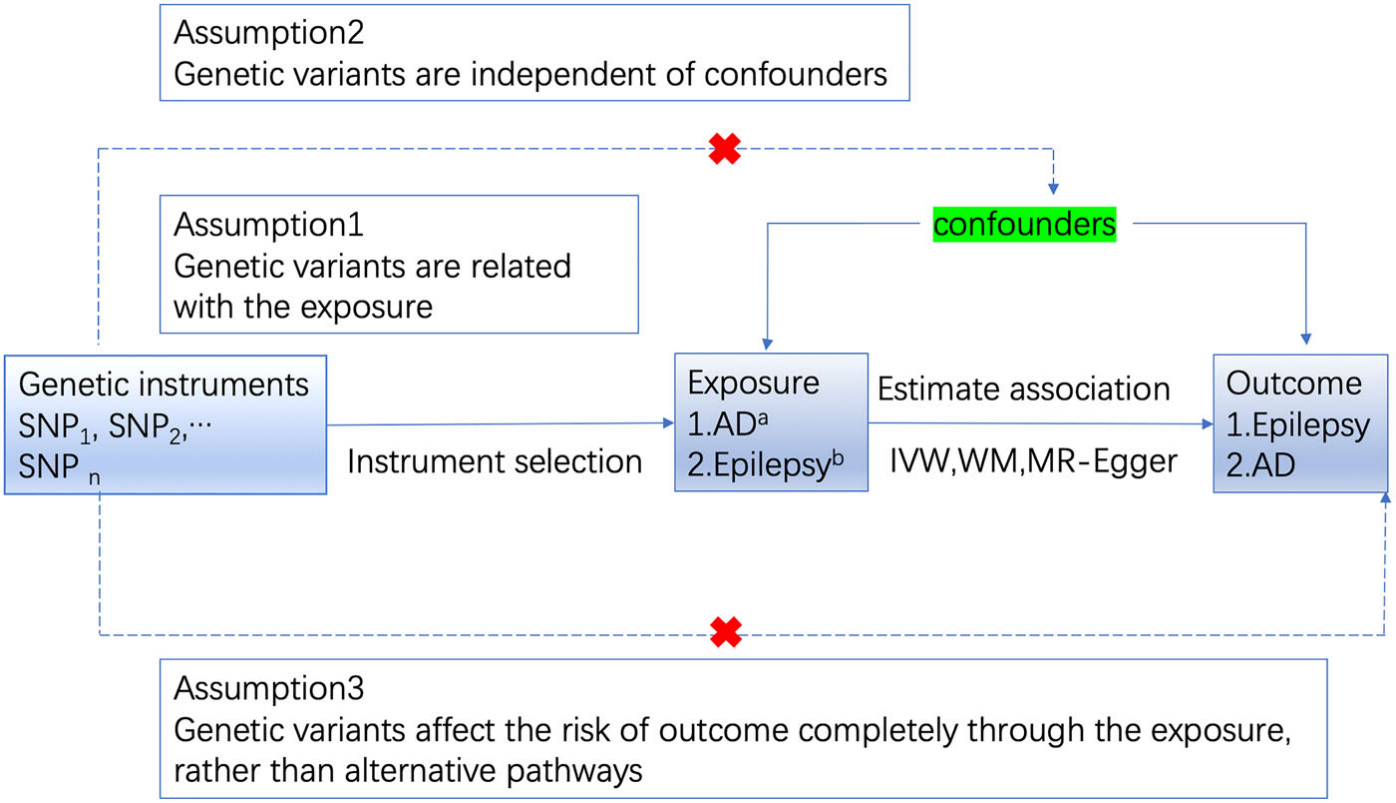
The process of the Mendelian randomization (MR) study. “a” Alzheimer's disease, Alzheimer's disease (late onset) (LOAD), “b” epilepsy (genome‐wide association studies [GWAS] ID: ieu‐b‐8), genetic generalized epilepsy (ieu‐b‐9), focal epilepsy (ieu‐b‐10), focal epilepsy‐documented lesion negative (ieu‐b‐11), juvenile absence epilepsy (ieu‐b‐12), childhood absence epilepsy (ieu‐b‐13), focal epilepsy‐documented hippocampal sclerosis (ieu‐b‐14), focal epilepsy‐documented lesions other than hippocampal sclerosis (ieu‐b‐15), generalized epilepsy with tonic–clonic seizures (ieu‐b‐16), and juvenile myoclonic epilepsy (ieu‐b‐17). SNP, single‐nucleotide polymorphism.

The relationship between AD and epilepsy has long intrigued researchers and clinicians, and there is evidence to suggest an association between the two conditions (Giorgi et al., 2020). However, most studies on this topic have been cross‐sectional or retrospective, with few prospective studies available. Mendelian randomization (MR) is a research approach that is similar in concept to a randomized controlled trial and can be used to investigate the causality of biomarkers in disease etiology based on aggregated data from genome‐wide association studies (GWAS) (Evans & Smith, 2015; Smith & Ebrahim, 2003). MR is based on three principal assumptions, which have been widely described in recent studies (Bowden et al., 2016). The first assumption is that the genetic variants used as instrumental variables (IVs) should be correlated with the exposure. The second assumption is that IVs should not be associated with confounding factors. The third assumption is that IVs should affect the risk of the outcome only through exposure. The second and third assumptions are known jointly as independence from pleiotropy (Davey Smith & Hemani, 2014; Pierce & Burgess, 2013) (Figure 1).

In recent years, large‐scale GWAS identified some genetic variants of AD and epilepsy. The large‐scale GWAS datasets provide enormous support for investigating the potential genetic association between AD and epilepsy risk by MR analytical method. Hence, we performed a 2‐sample MR study to clarify the causality and causal direction between AD and epilepsy, as well as the reverse direction.

## 2 MATERIALS AND METHODS

### 2.1 IV selection

This study is based on individuals of European ancestry from the IEU GWAS database (https://gwas.mrcieu.ac.uk/). All data were extracted from the IEU GWAS database. The AD GWAS data in this database included an AD (53,042 unique individuals who were either diagnosed with AD or AD‐by‐proxy who reported a parent or sibling having AD, and 355,900 controls) (Schwartzentruber et al., 2021) and LOAD (Lambert et al., 2013) (17,008 LOAD cases and 37,154 controls). AD‐by‐proxy, using parental diagnoses, demonstrated a strong genetic correlation with AD.

The GWAS data in this database included epilepsy, genetic generalized epilepsy, focal epilepsy, focal epilepsy‐documented lesion negative, juvenile absence epilepsy, childhood absence epilepsy, focal epilepsy‐documented hippocampal sclerosis (HS), focal epilepsy‐documented lesions other than HS, generalized epilepsy with tonic–clonic seizures, and juvenile myoclonic epilepsy based on the ILAE classification position paper on epilepsies (International League Against Epilepsy Consortium on Complex Epilepsies, 2018).

Details of the AD and epilepsy databases are provided in Table 1.

**TABLE 1:** Details of included database of Alzheimer's disease (AD) cohort and epilepsy cohort.

GWAS ID	Trait	Study	Sample size	Population
ebi‐a‐GCST90012878	Alzheimer's disease	PMID 33589840	408,942 (case 53,042, control 355,900)	European
ebi‐a‐GCST002245	Alzheimer's disease (late onset) (LOAD)	PMID 24162737	55,134 (case 17,008, control 37,154)	European
Ieu‐b‐8	Epilepsy	PMID 30531953	44,889	Mixed
Ieu‐b‐9	Generalized epilepsy	PMID 30531953	33,446 (case 3769, control 29,677)	Mixed
Ieu‐b‐10	Focal epilepsy	PMID 30531953	39,348 (case 9671, control 29,677)	Mixed
Ieu‐b‐11	Focal epilepsy‐documented lesion negative	PMID 30531953	39,393 (case 2716, control 29,677)	Mixed
Ieu‐b‐12	Juvenile absence epilepsy	PMID 30531953	30,092 (case 415, control 29,677)	Mixed
Ieu‐b‐13	Childhood absence epilepsy	PMID 30531953	30,470 (case 793, control 29,677)	Mixed
Ieu‐b‐14	Focal epilepsy‐documented hippocampal sclerosis	PMID 30531953	30,480 (case 803, control 29,677)	Mixed
Ieu‐b‐15	Focal epilepsy‐documented lesion other than	PMID 30531953	32,747 (case 3070, control 29,677)	Mixed
Ieu‐b‐16	Generalized epilepsy with tonic–clonic seizures	PMID 30531953	29,905 (case 228, control 29,677)	Mixed
Ieu‐b‐17	Juvenile myoclonic epilepsy	PMID 30531953	30,858 (case 1181, control 29,677)	Mixed

We selected single nucleotide polymorphisms (SNPs) that achieved a genome‐wide significance threshold (*p* < 5E − 08) for further analysis. These variants were then clumped together based on the linkage disequilibrium (LD) structure from the 1000 Genomes Project. Index SNPs, which had an LD (*R*2) of less than .01 with any other associated SNP within a 5000 kb region, were retained based on their minimum *p* value (Giorgi et al., 2020).

The current study is a secondary analysis conducted using publicly available data. Ethical approval was obtained for each of the original GWAS studies.

### 2.2 Mendelian randomization study

In the forward direction, we investigated whether AD (exposure) causally influences epilepsy (outcome). As outcome variables, we selected epilepsy (GWAS ID: ieu‐b‐8), genetic generalized epilepsy (ieu‐b‐9), focal epilepsy (ieu‐b‐10), focal epilepsy‐documented lesion negative (ieu‐b‐11), focal epilepsy‐documented HS (ieu‐b‐14), focal epilepsy‐documented lesions other than HS (ieu‐b‐15), and generalized epilepsy with tonic–clonic seizures (ieu‐b‐16) but did not include epilepsy syndrome with onset in childhood or adolescence.

In the reverse direction, our analysis aimed to determine whether genetic predisposition to epilepsy (exposure) causally affects AD (outcome).

In the validation step, we utilized the latest available AD dataset (AD and AD‐by‐proxy = 85,934, *N*_control_ = 401,577) to verify our results (Bellenguez et al., 2022). The dataset was accepted from GWAS catalog (GCST90027158).

The study used several statistical analyses to estimate the causal effect between exposure and outcome. The main analysis used the inverse variance‐weighted (IVW) method, which was used to calculate an overall causal effect between the SNP exposure and outcome (Burgess et al., 2017).

The weighted median method was used as a sensitivity analysis. The study also used MR‐Egger regression to evaluate directional pleiotropy. MR‐PRESSO was used to identify outlier‐adjusted estimates with a significant global test *p* value less than .05. The study performed the Q test in the IVW test to evaluate the heterogeneity of results (Burgess & Thompson, 2017; Verbanck et al., 2018). “Leave‐one‐out” analyses were also conducted to estimate the causal effect of outlying IVs by stepwise removing each IV from the MR analysis.

All statistical analyses were conducted using the R statistical software version 4.1.3 with the R packages “TwoSample MR” and “MR‐PRESSO.” The study considered *p* < .05 as statistically significant.

## 3 RESULTS

For the forward direction analysis, we selected 22 SNPs for LOAD and 23 SNPs for AD based on our established criteria, and detailed information on all included SNPs can be found in Supporting Information Excel sheet 1. Using the IVW method, we found a significant positive effect of LOAD on focal epilepsy (GWAS ID: ieu‐b‐10) (OR_IVW_ = 1.079, *p*_IVW_ = .013) (Table 2), but no significant effects of AD or LOAD on epilepsy or its subtypes, except for focal epilepsy in the case of LOAD. Supporting Information Excel sheet 2 provides further details on all results.

**TABLE 2:** The result of Alzheimer's disease (late onset) (LOAD) on focal epilepsy, focal epilepsy‐documented hippocampal sclerosis (Focal epilepsy_HS) on AD.

		IVW		WME		MR‐Egger	
Exposure	Outcome	OR	*p*	OR	*p*	OR	*p*
LOAD	Focal epilepsy	1.079	.013	1.050	.203	1.002	.987
Focal epilepsy_HS	Alzheimer's disease	1.152	.017	NA	NA	NA	NA

Abbreviations: IVW, inverse variance‐weighted; MR, Mendelian randomization; WME, Weighted median.

For the reverse direction analysis, we included 3 SNPs for epilepsy (ieu‐b‐8), 11 SNPs for generalized epilepsy (ieu‐b‐9), and 2 SNPs for focal epilepsy‐documented HS (ieu‐b‐14) based on our criteria, as detailed in Supporting Information Excel sheet 1. Other epilepsy cohorts were not included in this analysis due to a lack of sufficient SNPs according to the IV selection criteria. Using the IVW method, we found a significant positive effect of focal epilepsy‐documented HS (ieu‐b‐14) on AD (OR_IVW_ = 1.152, *p*_IVW_ = .017) (Table 2), but no significant effects of other epilepsy subtypes on AD or LOAD. Supporting Information Excel sheet 2 provides additional information on these findings.

For the validation analysis, the MR using the IVW method, we found a significant two‐way causality between AD and focal epilepsy‐documented HS. We found a significant positive effect of AD on focal epilepsy‐documented HS (ieu‐b‐14) (OR_IVW_ = 1.012, *p*_IVW_ = .023), a significant positive effect of focal epilepsy‐documented HS (ieu‐b‐14) on AD (OR_IVW_ = 3.994, *p*_IVW_ = .027).

### 3.1 Sensitivity analyses

**FIGURE 2:**
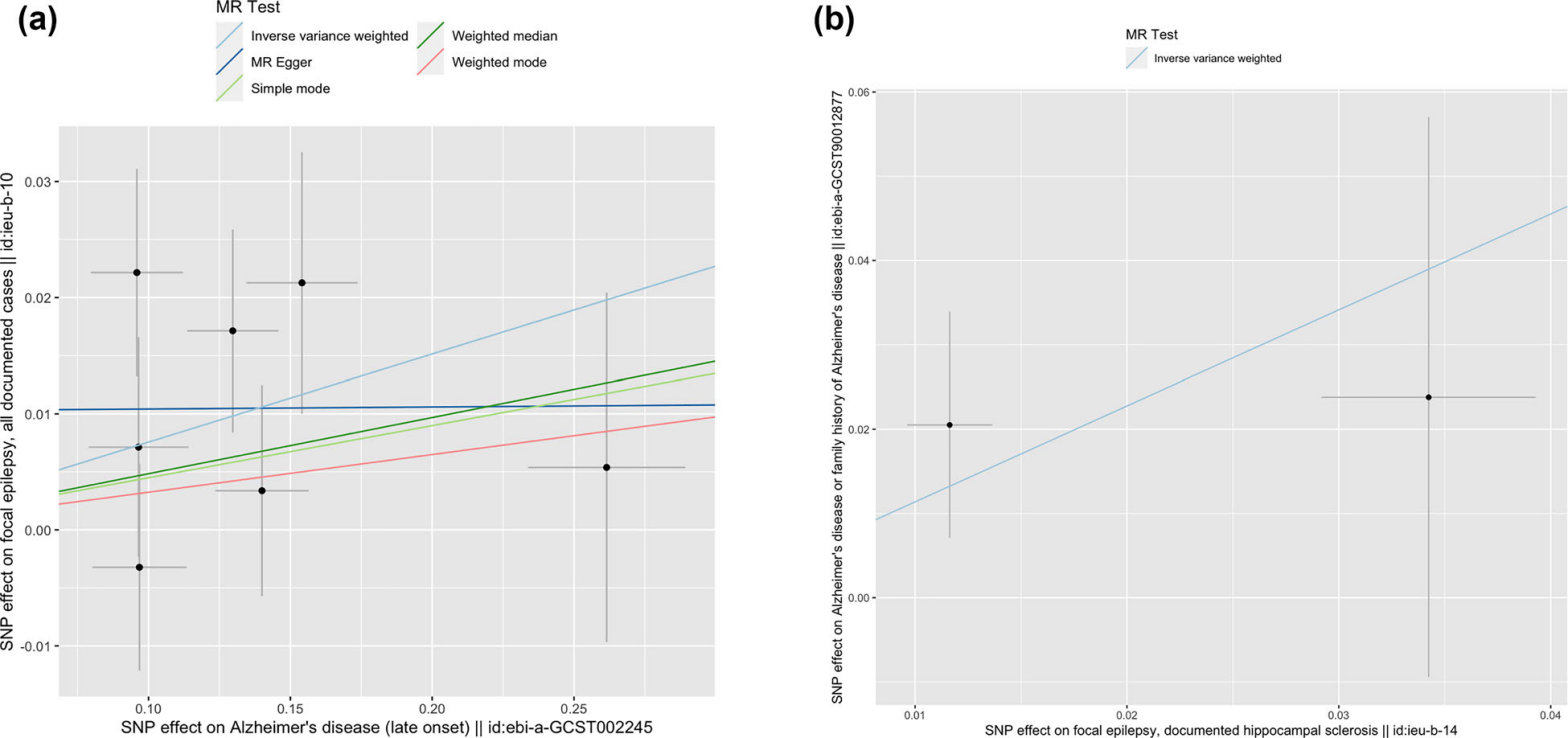
Scatter plot of potential effects of Alzheimer's disease (AD) and epilepsy. The slope of each line corresponds to the estimated Mendelian randomization (MR) effect of each method: (A) scatter plot of AD (late onset) (LOAD) on focal epilepsy (ieu‐b‐10), (B) scatter plot of focal epilepsy‐documented hippocampal sclerosis (ieu‐b‐14) on AD. IVW, inverse variance‐weighted; MR‐Egger, Mendelian randomization‐Egger; SNP single nucleotide polymorphisms.

**FIGURE 3:**
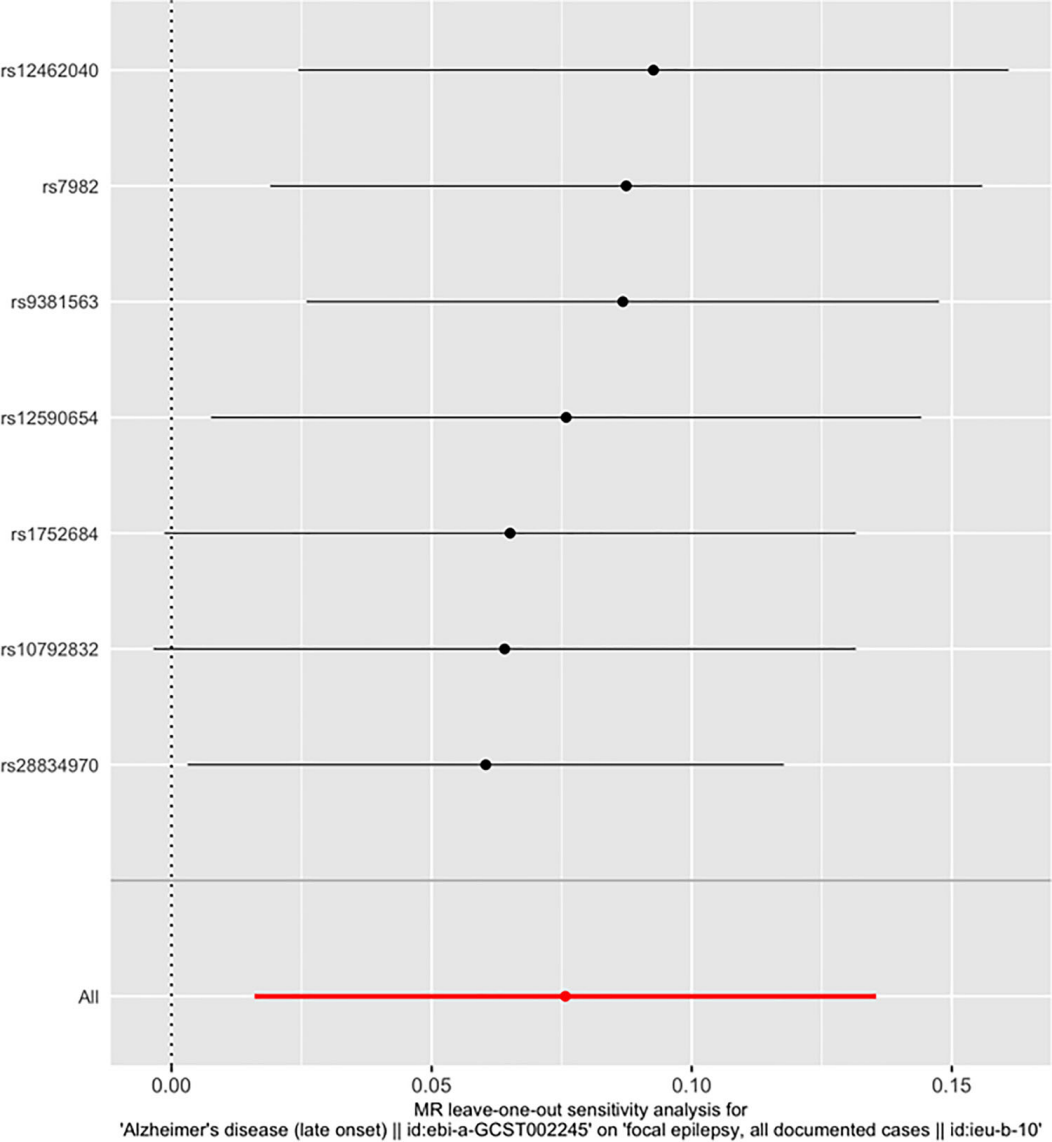
Leave‐one‐out sensitivity analysis for late‐onset Alzheimer's disease (LOAD) and focal epilepsy. Each row (black lines) represents the result after the removal of the corresponding single nucleotide polymorphism (SNP), and the overall effect is shown at the bottom (redline).

Sensitivity analyses (weighted median, simple median, MR‐Egger, and MR‐PRESSO) showed consistent results with the main random‐effect IVW. For all analyses, there was no strong evidence of directional pleiotropy from the MR‐Egger regression intercepts. The results did not reveal significant heterogeneity, except in the validation analysis between AD and epilepsy/focal epilepsy/ focal epilepsy‐documented lesions other than HS. The results of the sensitivity analyses are shown in Table 3. The results were similar after removing single SNPs in the leave‐one‐out analysis, suggesting that no single SNP had an exorbitant influence on the overall estimates (Figures 2 and 3).

**TABLE 3:** *p* Value of Mendelian randomization (MR) heterogeneity test, pleiotropy test, MR‐PRESSO global test.

				Heterogeneity test			MR‐PRESSO global test
Exposure	Outcome (ID)	*R*2	*F*	IVW	MR‐Egger	Pleiotropy test	
LOAD	Focal epilepsy	.0086	55.67	.3060	.2688	.4834	.349
Focal epilepsy_HS	AD	.0026	39.77	.4783	NA	NA	NA

Abbreviations: AD, Alzheimer's disease; IVW, inverse variance‐weighted; LOAD, late‐onset AD.

## 4 DISCUSSION

Based on our MR study, focal epilepsy‐documented HS was found to increase the risk of AD, and at the same time, LOAD was identified as a risk factor for focal epilepsy. Through validation analysis, our conclusion that focal epilepsy‐documented HS and AD have a causal relationship was confirmed, and it was further demonstrated that they have a bidirectional causal relationship. Sensitivity analyses of the all above cohort MR studies showed no genetic heterogeneity or pleiotropy, indicating that our results are robust. Our study's results provide strong evidence supporting the relationship between focal epilepsy‐documented HS and AD.

In a recent MR study conducted by Fang et al. published in the Neurology journal, similar to our study on AD and epilepsy, they concluded that AD increases the risk of HS‐related focal epilepsy and generalized epilepsy (Fang et al., 2023). The epilepsy cohorts used in our study and Fang's study are the same, and they both come from LIAE. In Fang's study, the discovery process utilized the AD cohort, and for validation utilized the LOAD cohort. In our study, we included the AD cohort (which Fang's study did not include) and the LOAD cohort (similar to Fang's inclusion). For validation, we also included the AD cohort (AD and proxy cases) (similar to Fang's inclusion). However, AD and LOAD exposure cohorts were different in the two studies (Table 4). Our study primarily employed the IVW method, whereas their study utilized the GSMR method (Zhu et al., 2018). The convergence of similar conclusions from different methodological approaches adds credibility to the findings. Both studies found consistent correlations and *p* values less than .05 in the relationship between HS and epilepsy (exposure) and AD (outcome).

**TABLE 4:** The genome‐wide association studies (GWAS) datasets were included in Fang's study and in my study.

		Trait	Study	Sample size (*N*_case_/*N*_control_)
Fang. et al.	Discover	AD (AD and proxy cases)	Bellenguez et al. (2022)	111,326/677,663 (exposure) 85,934/401,577 (outcome)
		Epilepsy	International League Against Epilepsy Consortium on Complex Epilepsies (2018)	15,212/29,677
	Validation	LOAD	Lambert et al. (2013)	25,580/48,466 (exposure) 17,008/37,154 (outcome)
Our	Discover	AD1 (AD and Proxy cases)	Schwartzentruber et al. (2021)	53,042/1,355,900
		LOAD	Lambert et al. (2013)	17,008/37,154
		Epilepsy	International League Against Epilepsy Consortium on Complex Epilepsies (2018)	15,212/29,677
	Validation	AD (AD and proxy cases)	Bellenguez et al. (2022)	85,934/401,577

Abbreviations: AD, Alzheimer's disease; LOAD, late‐onset AD.

Our study's findings are in line with previous research. A review published in Dement Neuropsychol in 2014 revealed that epilepsy is more frequent among AD patients (Miranda & Brucki, 2014). One study identified 19 articles out of 3043 citations that were relevant to the correlation between dementia and epilepsy. The study conducted by Zelano and published in the European Journal of Neurology is the largest study to date (*N*_case_ = 81,192, *N*_control_ = 223,933), and it provides evidence that individuals with early‐onset AD are at a higher risk of developing epilepsy compared to individuals experiencing normal aging (Zelano et al., 2020). In 2020, Johnson et al. published a study in the journal Neurology, which included 9033 participants from the ARIC (The Atherosclerosis Risk in Communities) cohort. They concluded that the risk of incident dementia is significantly higher in individuals with late‐onset epilepsy (Johnson et al., 2020).

The histopathologic finding of HS can be seen in association with AD and temporal lobe epilepsy. AD is characterized by significant pathological changes in the temporal lobe and hippocampus, which are the primary sites of Aβ and Tau accumulation. Recent studies indicate that phosphorylated Tau protein in the hippocampus is associated with cognitive decline in temporal lobe epilepsy (Tai et al., 2016). Additionally, amyloid ß pathological alterations have been observed in both temporal lobe epilepsy and AD (Price et al., 2001; Scharfman, 2007; Ziyatdinova et al., 2011). The hallmark of AD senile plaques was also found in epilepsy patients (Buda et al., 2009). Both epilepsy and AD involve various pathophysiological events, such as changes in synaptic transmission, heightened network hyperexcitability, decreased activity of GABAergic interneurons, modifications in voltage‐gated ion channels, cytoskeletal dysfunction, increased amyloid‐β expression, cerebrovascular changes, neuroinflammation, and oxidative stress (Cheng et al., 2020; Johnson et al., 2020; Johnson et al., 2020; Vossel et al., 2017). Shared mechanisms between the two conditions include tau hyperphosphorylation and abnormalities in certain enzymes like GSK‐3β and caspases (Toral‐Rios et al., 2020). Amyloid‐β plays a significant role in altering calcium homeostasis in neurons and glia, leading to changes in neuronal structure and impaired neurotransmitter systems. It can induce a decline in sodium channel activity, affecting parvalbumin‐positive interneurons and contributing to epileptic seizures. Additionally, amyloid‐β oligomers can cause synaptic damage, impaired synaptic plasticity, and altered coordinated network activity (Sánchez et al., 2018). Seizures induce a process of tau hyperphosphorylation and amyloidogenic marker expression in the hippocampus, which is accompanied by the dysregulation of inflammatory responses over time (Canet et al., 2022; Sánchez et al., 2018). These provide compelling evidence for the causal relationship between AD and focal epilepsy with HS.

There have been numerous discoveries of intricate connections between AD and epilepsy at the genetic level. The genetic basis of AD and epilepsy has also been investigated (Wang et al., 2021). Mutations in the *PSEN1* gene are associated with both conditions (Larner, 2011). The study proposed that changes in the genes encoding GSK3β and Tau are genetic factors leading to the development of AD and temporal lobe epilepsy (Toral‐Rios et al., 2020).

Our MR study provides robust evidence supporting a bidirectional relationship between epilepsy and AD. Our study is strengthened by the use of large‐scale GWAS summary data and sensitivity analyses, which confirmed the robustness of our results. The MR design employed in our study is particularly advantageous in investigating causal relationships. This approach allowed us to minimize potential biases and confounding factors in observational studies. In summary, the use of GWAS‐derived genetic risk factors, combined with the MR design, the IVW statistic method, strengthens the validity of our findings and provides valuable insights into the association between AD and epilepsy.

However, there are also some limitations to our study. First, although we used genetic variants with strong associations as IVs, there is still a possibility of weak instrument bias. Second, our study mainly focuses on individuals of European ancestry, and therefore, our findings may not be generalizable to non‐European populations. Third, the sample size for specific epilepsy subtypes was relatively small.

## 5 CONCLUSION

We used a 2‐sample MR to detect the bidirectional relationship between AD and epilepsy, and our results support that focal epilepsy‐documented HS increases the risk of AD.

## AUTHOR CONTRIBUTIONS

**Lianping Xu**: Conceptualization; Data curation; Formal analysis; Investigation; Methodology; Project administration; Resources; Software; Supervision; Validation; Visualization; Writing‐original draft; Writing‐review & editing. **Qun Wang**: Funding acquisition; Supervision; Validation; Writing‐review & editing.

## CONFLICT OF INTEREST STATEMENT

The authors declare that they have no conflicts of interest.

### PEER REVIEW

The peer review history for this article is available at https://publons.com/publon/10.1002/brb3.3221.

## Supporting information

Supporting Information

## Data Availability

Summary‐level data for the exposures and outcomes were drawn from the Ieu Open Gwas Project at https://gwas.mrcieu.ac.uk/. The AD dataset for validation was accepted from GWAS catalog at https://www.ebi.ac.uk/gwas/.
